# Longitudinal Diaphragm Ultrasound Assessment Across Respiratory Support Modalities in Critically Ill Infants and Children: A Prospective Cohort Study

**DOI:** 10.3390/children13081104

**Published:** 2026-08-18

**Authors:** Kubra Boydag Guvenc, Ebru Guney Sahin, Idris Abdullah Yılmaz, Fatih Varol, Cansu Durak

**Affiliations:** Department of Pediatric Intensive Care, Sancaktepe Sehit Prof. Dr. Ilhan Varank Training and Research Hospital, University of Health Sciences, Istanbul 34785, Turkey

**Keywords:** diaphragm ultrasonography, pediatric intensive care, respiratory support, respiratory muscle function, noninvasive ventilation, mechanical ventilation

## Abstract

**Highlights:**

**What are the main findings?**

- No statistically significant longitudinal changes in diaphragm ultrasound parameters were detected during short-term respiratory support.
- No statistically significant between-group differences were detected across HFNC, nasal NIV, mask NIV, and IMV.

**What are the implications of the main findings?**

- Respiratory support modality alone may be insufficient to explain early diaphragm ultrasound findings.
- Diaphragm ultrasonography should be interpreted alongside the overall clinical assessment rather than used as an isolated prognostic tool.

**Abstract:**

**Background/Objectives**: Diaphragm ultrasonography enables noninvasive bedside assessment of diaphragmatic structure and function. However, longitudinal changes in diaphragm ultrasound parameters across different respiratory support modalities remain insufficiently characterized in critically ill children. This exploratory study aimed to evaluate serial changes in diaphragm ultrasound parameters and to explore whether these changes differed according to the initial respiratory support modality or showed a relationship with respiratory outcomes. **Methods**: This prospective, single-center observational cohort study included children aged 1 month to 18 years who required respiratory support for acute respiratory failure between March 2025 and March 2026. Diaphragm excursion (DE), end-expiratory diaphragm thickness (DTee), end-inspiratory diaphragm thickness (DTei), and diaphragm thickening fraction (TFdi) were measured at baseline, 48 h, and immediately before discontinuation of respiratory support. Patients were grouped according to the initial respiratory support modality: high-flow nasal cannula, nasal noninvasive ventilation, mask noninvasive ventilation, or invasive mechanical ventilation. **Results**: A total of 100 children were included; the median age was 4 months, and 66% were male. No statistically significant longitudinal changes were observed in DE (*p* = 0.210), TFdi (*p* = 0.850), DTee (*p* = 0.492), or DTei (*p* = 0.467). Baseline values and longitudinal percentage changes did not differ significantly among the four respiratory support groups. No statistically significant relationship was observed between serial changes in DE, TFdi, or DTee and total respiratory support duration. **Conclusions**: No statistically significant longitudinal changes in diaphragm ultrasound parameters were detected during short-term respiratory support, and no statistically significant differences were identified among the initial respiratory support groups. These findings should not be interpreted as demonstrating physiological equivalence between respiratory support modalities. Serial diaphragm ultrasonography should be interpreted within the broader clinical context rather than as an isolated prognostic tool.

## 1. Introduction

Acute respiratory failure is one of the leading reasons for admission to pediatric intensive care units (PICUs) and frequently necessitates respiratory support ranging from high-flow nasal cannula (HFNC) therapy to invasive mechanical ventilation (IMV) [1,2]. The diaphragm, as the principal muscle of inspiration, plays a central role in maintaining alveolar ventilation [3]. Accordingly, diaphragmatic dysfunction may reduce respiratory reserve, contribute to ventilatory failure, and prolong the need for respiratory support. In recent years, diaphragm ultrasonography has emerged as a valuable bedside imaging modality that enables noninvasive, repeatable, and real-time assessment of diaphragmatic structure and function in critically ill children [4,5].

The effect of respiratory support on the diaphragm is determined largely by the balance between respiratory muscle loading and unloading [6]. HFNC and noninvasive ventilation (NIV) generally preserve spontaneous respiratory effort, whereas IMV may substantially reduce diaphragmatic workload depending on the level of ventilatory assistance [5,7]. Insufficient unloading may expose the diaphragm to excessive inspiratory effort and fatigue, whereas excessive unloading and suppression of spontaneous breathing have been associated with ventilator-induced diaphragm dysfunction characterized by muscle atrophy and impaired contractility [7]. Consequently, different respiratory support modalities may influence diaphragm structure and function differently throughout the course of critical illness.

Previous adult and pediatric studies have evaluated ultrasonographic parameters, including diaphragm thickness (DT), diaphragm thickening fraction (TFdi), and diaphragm excursion (DE), as markers of diaphragmatic dysfunction and predictors of weaning outcomes in mechanically ventilated patients [5,7]. Pediatric studies have suggested that DT may decrease within the first few days of invasive mechanical ventilation [8], whereas functional indices such as DE and TFdi may be associated with successful liberation from respiratory support [9,10,11]. However, the available pediatric evidence remains limited. Most studies have focused exclusively on invasively ventilated patients, assessed a single ultrasound parameter, or performed measurements only around the time of extubation. In addition, considerable heterogeneity in study populations, ultrasound protocols, and outcome definitions has limited comparisons across studies and reduced the generalizability of current evidence [12].

Consequently, comprehensive longitudinal evaluations incorporating DE, DT, and TFdi across HFNC, NIV, and IMV remain scarce in pediatric critical care. We hypothesized that diaphragm ultrasound parameters might change over the course of respiratory support and that the magnitude or pattern of these longitudinal changes might differ according to the initial respiratory support modality. Therefore, this prospective exploratory study aimed to characterize longitudinal changes in diaphragm ultrasound parameters during respiratory support in critically ill infants and children and to explore whether these changes differed according to the initial respiratory support modality or showed a relationship with clinically relevant respiratory outcomes.

## 2. Materials and Methods

### 2.1. Study Design and Setting

This prospective, single-center observational cohort study was conducted in the Pediatric Intensive Care Unit of Sancaktepe Prof. Dr. İlhan Varank Training and Research Hospital, Türkiye, between March 2025 and March 2026. The study protocol was approved by the Institutional Ethics Committee (Approval No. 2025/38, 12 February 2025) and was conducted in accordance with the Declaration of Helsinki. Written informed consent was obtained from the parents or legal guardians of all participants before enrollment.

### 2.2. Study Population

Children aged 1 month to 18 years who required invasive or noninvasive respiratory support because of acute respiratory failure were prospectively screened for eligibility.

Eligible patients were prospectively enrolled if diaphragm ultrasonography could be performed within the first 6 h after initiation of respiratory support and serial ultrasound assessments were planned according to the study protocol. Patients in whom the planned serial assessments could not subsequently be completed because of early discontinuation of respiratory support, urgent clinical intervention, hemodynamic instability, transfer to another hospital or ward, or logistical limitations were excluded from the final longitudinal analysis. Urgent clinical intervention and hemodynamic instability were not prespecified clinical exclusion criteria; rather, these patients were excluded only when such conditions prevented the safe and reliable completion of the protocol-defined serial ultrasound assessments. For patients with more than one eligible PICU admission during the study period, only the first admission was included to preserve the independence of observations and to avoid disproportionate weighting of patients with recurrent admissions.

Patients with known or suspected diaphragmatic paralysis, neuromuscular disorders affecting respiratory muscle function, bronchopulmonary dysplasia, chronic ventilator dependence, chronic pulmonary diseases expected to alter diaphragm morphology or function, previous thoracic or upper abdominal surgery, or inadequate sonographic visualization preventing reliable diaphragm assessment were excluded.

This study was designed as an exploratory prospective cohort study. Although pediatric longitudinal studies of diaphragm ultrasonography were available, they focused predominantly on children receiving invasive mechanical ventilation and differed substantially in study populations, ultrasound parameters, assessment intervals, and outcome definitions. Therefore, directly comparable effect-size estimates across HFNC, NIV, and IMV were not available to support a reliable a priori sample-size calculation. Adult-derived effect estimates were also not considered directly transferable because of age-related differences in diaphragm morphology, chest-wall compliance, respiratory mechanics, and respiratory support practices. Accordingly, all consecutive patients meeting the eligibility criteria during the predefined study period were prospectively enrolled.

### 2.3. Respiratory Support Management

The choice of respiratory support modality, ventilator mode, interface, and ventilator settings was at the discretion of the attending pediatric intensivist and followed routine clinical practice in our PICU. Invasively ventilated patients were managed according to lung-protective ventilation principles consistent with contemporary pediatric critical care recommendations [1,2]. The strategy aimed to avoid excessive tidal volumes and inspiratory pressures while maintaining adequate oxygenation and ventilation. Tidal volume was individualized according to body weight and respiratory system mechanics, and PEEP and FiO_2_ were titrated according to oxygenation requirements, lung mechanics, and hemodynamic tolerance. Inspiratory pressures were limited as clinically feasible to minimize ventilator-induced lung injury. Ventilator mode and individual settings were adjusted according to respiratory mechanics, gas exchange, and clinical condition. The study protocol did not mandate fixed ventilator targets or a single ventilation mode, and no ventilator adjustments were performed specifically for study purposes.

HFNC, nasal-interface NIV, mask-interface NIV, and IMV were analyzed as four distinct exposure groups and were not pooled. Initial settings and subsequent adjustments, including gas flow, inspiratory and expiratory pressures, and fraction of inspired oxygen (FiO_2_), were individualized according to each patient’s clinical condition and modified throughout treatment according to clinical response. Patients remained within their initial respiratory support group throughout the relevant respiratory support episode. Brief scheduled interruptions of noninvasive respiratory support, during which supplemental oxygen was administered via a reservoir mask for 1 h every 3 h, were considered temporary treatment breaks and not transitions between respiratory support modalities. Diaphragm ultrasound assessments were performed while patients were receiving their ongoing respiratory support modality rather than during these scheduled breaks.

Patients requiring invasive mechanical ventilation received pressure-controlled ventilation or pressure-regulated volume-targeted ventilation according to the treating physician’s judgment and respiratory mechanics. High-frequency oscillatory ventilation was not used in any patient included in the study. Ventilator settings, including peak inspiratory pressure (PIP), positive end-expiratory pressure (PEEP), mean airway pressure (MAP), tidal volume indexed to body weight (mL/kg), and FiO_2_, were recorded simultaneously with each diaphragm ultrasound assessment. No protocol-driven adjustments were performed for study purposes.

NIV failure was defined as the need for endotracheal intubation following deterioration despite noninvasive respiratory support. Extubation failure was defined as reintubation within 48 h after planned extubation. Planned post-extubation NIV was not considered extubation failure.

Total respiratory support duration was defined as the interval from initiation of respiratory support until complete discontinuation of all respiratory support modalities.

### 2.4. Clinical Data Collection

Demographic and clinical characteristics were prospectively recorded at enrollment, including age, sex, body weight, primary diagnosis, presence of comorbidities, initial respiratory support modality, total respiratory support duration, NIV failure, and extubation failure.

### 2.5. Diaphragm Ultrasound Protocol

Diaphragm ultrasonography was performed by a single pediatric intensivist experienced in diaphragm ultrasound using a Samsung HM70 EVO ultrasound system (Samsung Medison, Seoul, Republic of Korea). All serial examinations were performed by the same operator to minimize interobserver variability.

Patients were examined in the supine or 30–45° semi-recumbent position according to clinical tolerance. Whenever clinically feasible, serial examinations in the same patient were performed in the same body position.

Ultrasound examinations were performed under the prevailing respiratory support conditions. In invasively ventilated patients, measurements were obtained after stabilization of ventilator settings, whereas in patients receiving noninvasive respiratory support, examinations were performed while support was ongoing.

The study protocol did not standardize ventilator mode, spontaneous respiratory activity, the level of ventilatory assistance, sedation depth, or the use of neuromuscular blockade during ultrasound acquisition, because the objective was to evaluate diaphragm ultrasound findings under routine clinical conditions.

Active neuromuscular blockade was present during the T1 and T2 assessments in four patients and during the T1 assessment only in one patient. Therefore, a sensitivity analysis was performed after excluding patients who underwent at least one protocol-defined ultrasound assessment during active neuromuscular blockade.

The right hemidiaphragm was evaluated in all patients, as the liver provides a stable acoustic window that facilitates superior image quality and improves measurement reproducibility; accordingly, right-sided assessment has been recommended for standardized diaphragm ultrasonography in previous studies [13].

To assess interobserver reliability, diaphragm ultrasound examinations were independently repeated in 10 patients selected by systematic sampling (every tenth consecutively enrolled patient starting from the fifth enrolled patient). Both observers independently performed the examinations under the same clinical conditions and were blinded to each other’s measurements. Agreement was assessed using a two-way random-effects, absolute-agreement, single-measure intraclass correlation coefficient (ICC) with corresponding 95% confidence intervals.

### 2.6. Ultrasound Measurements

#### 2.6.1. Diaphragm Excursion

DE was measured using a 2–5 MHz convex transducer placed in the right subcostal region. After identification of the diaphragm in B-mode, M-mode was used to record diaphragmatic motion. DE was defined as the craniocaudal displacement of the diaphragm from end-expiration to peak inspiration and was expressed in centimeters. Three consecutive respiratory cycles were recorded, and the mean value was used for analysis [14].

#### 2.6.2. Diaphragm Thickness and Thickening Fraction

DT was assessed in the zone of apposition using a 6–13 MHz linear transducer positioned between the eighth and tenth intercostal spaces along the anterior-to-midaxillary line [15].

End-expiratory diaphragm thickness (DTee) and end-inspiratory diaphragm thickness (DTei) were measured as the distance between the inner borders of the pleural and peritoneal membranes, excluding the membranes themselves [16]. Measurements were obtained from three consecutive respiratory cycles under the prevailing ventilatory conditions, and the mean value was used for analysis.

TFdi was calculated according to the following equation:

(1) TFdi (%) = (DTei − DTee)/DTee × 100

#### 2.6.3. Assessment Time Points and Change Calculations

Diaphragm ultrasound examinations were performed at predefined time points throughout respiratory support.

The baseline examination (T1) was obtained within 6 h after initiation of respiratory support. The second examination (T2) was performed 48 h after respiratory support initiation. The third examination (T3) was obtained immediately before discontinuation of respiratory support. Baseline values corresponded to T1.

Early percentage change was calculated between T1 and T2.

Overall percentage change was calculated between T1 and the final protocol-defined ultrasound examination obtained immediately before complete discontinuation of respiratory support.

Percentage change was calculated as

(2) %Δ = (Follow-up − Baseline)/Baseline × 100

### 2.7. Outcome Measures

The primary outcome was the longitudinal change in diaphragm ultrasound parameters during respiratory support, including DE, TFdi, DTee, and DTei.

Secondary outcomes included comparisons of baseline values and longitudinal percentage changes according to the initial respiratory support modality.

Exploratory analyses evaluated the associations between serial percentage changes in DE, TFdi, and DTee and clinically relevant respiratory outcomes, including total respiratory support duration, NIV failure, and extubation failure, where the number of events permitted meaningful statistical evaluation. These analyses were considered hypothesis-generating.

### 2.8. Statistical Analysis

Statistical analyses were performed using IBM SPSS Statistics for Windows, version 22.0 (IBM Corp., Armonk, NY, USA).

The distribution of continuous variables was assessed using the Shapiro–Wilk test together with visual inspection of histograms and Q–Q plots. Continuous variables were summarized as median and interquartile range (IQR) because most variables were not normally distributed. For contextual comparison with published age-specific reference data reported as mean ± SD, baseline DE in the predominant age subgroup was additionally summarized as mean ± SD. Categorical variables were presented as frequencies and percentages.

Comparisons of baseline diaphragm ultrasound parameters and longitudinal percentage changes among the four initial respiratory support modalities (HFNC, nasal NIV, mask NIV, and IMV) were performed using the Kruskal–Wallis test. Effect sizes for between-group comparisons were estimated using epsilon squared (ε^2^) and are provided in the Supplementary Material.

Longitudinal changes in diaphragm ultrasound measurements obtained at baseline, 48 h, and the end of respiratory support were evaluated using the Friedman test. All 100 patients had evaluable measurements at all three predefined time points and were included in the primary longitudinal analyses.

Relationships between serial percentage changes in diaphragm ultrasound parameters and total respiratory support duration were explored using Spearman’s rank correlation coefficient (ρ).

Exploratory comparisons according to NIV failure and extubation failure were performed using the Mann–Whitney U test, where the number of events permitted meaningful statistical evaluation.

Interobserver reliability of diaphragm ultrasound measurements was assessed using a two-way random-effects, absolute-agreement, single-measure intraclass correlation coefficient (ICC) with corresponding 95% confidence intervals.

Within the IMV subgroup, a sensitivity analysis of longitudinal diaphragm ultrasound measurements was performed after excluding patients who underwent at least one protocol-defined ultrasound assessment during active neuromuscular blockade.

All statistical tests were two-sided, and a *p* value <0.05 was considered statistically significant. Owing to the exploratory nature of the secondary analyses, no adjustment for multiple comparisons was performed.

## 3. Results

### 3.1. Patient Characteristics

**Figure 1:**
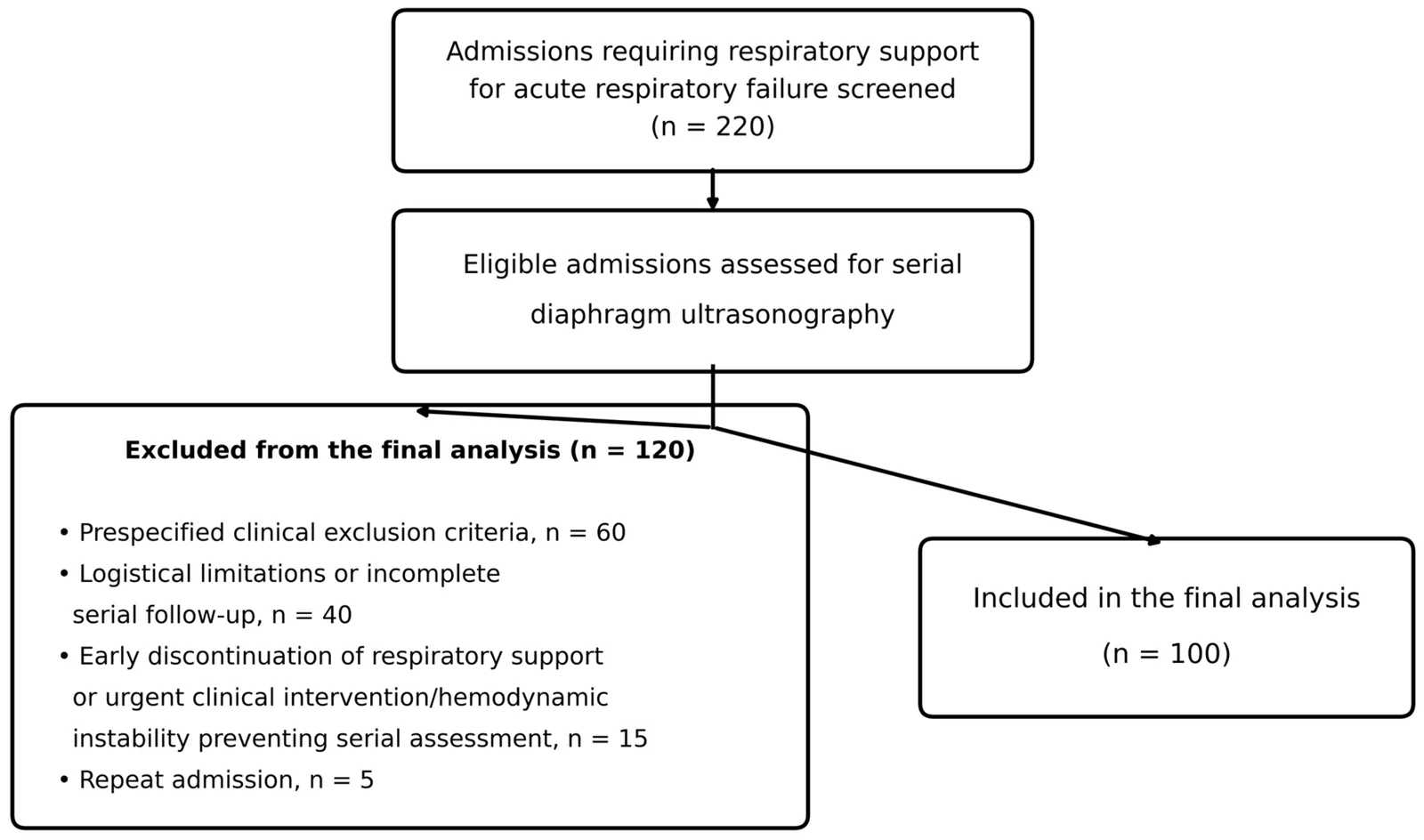
Flow diagram of patient screening and enrollment.

Of 220 admissions requiring respiratory support for acute respiratory failure screened during the study period, 100 patients were included in the final analytic cohort (Figure 1). The numbers and principal reasons for exclusion from the final analysis are detailed in Figure 1.

The median age was 4 months (IQR, 2–24), and 66 (66.0%) were male. Bronchiolitis or pneumonia was the most frequent admission diagnosis (89.0%), followed by neurological diseases (5.0%), trauma (4.0%), and cardiac diseases (2.0%). Six patients (6.0%) had at least one underlying comorbidity. Initial respiratory support consisted of HFNC in 31 patients (31.0%), nasal NIV in 17 (17.0%), mask NIV in 24 (24.0%), and IMV in 28 (28.0%). The median total duration of respiratory support was 3.5 days (IQR, 2.0–5.0) for the overall cohort. According to the initial respiratory support modality, the median duration was 2.0 days (IQR, 2.0–4.0) in the HFNC group, 4.0 days (IQR, 2.0–5.0) in the nasal NIV group, 4.0 days (IQR, 2.0–5.0) in the mask NIV group, and 8.0 days (IQR, 2.9–14.8) in the IMV group. Neuromuscular blockade was administered in 5 of the 28 invasively ventilated patients (17.8%). Children aged 1 month–2 years constituted 77% of the cohort. In this predominant age subgroup, mean baseline DE was 0.88 ± 0.28 cm. Baseline patient characteristics are presented in Table 1.

### 3.2. Diaphragm Ultrasound Parameters According to the Initial Respiratory Support Modality

No statistically significant differences in baseline diaphragm ultrasound measurements were detected across the four initial respiratory support groups. Specifically, DE, DTee, DTei, and TFdi did not differ significantly according to the initial respiratory support modality (all *p* > 0.05) (Table 2).

No statistically significant between-group differences were detected in early percentage changes for any diaphragm ultrasound parameter. Similarly, no statistically significant between-group differences were detected in overall percentage changes from baseline to the end of respiratory support (all *p* > 0.05) (Table 2). Effect size analysis demonstrated negligible between-group effects for most comparisons, with small effect sizes observed only for early changes in TFdi and DTei (Supplementary Table S3).

### 3.3. Longitudinal Changes in Diaphragm Ultrasound Measurements

**Figure 2:**
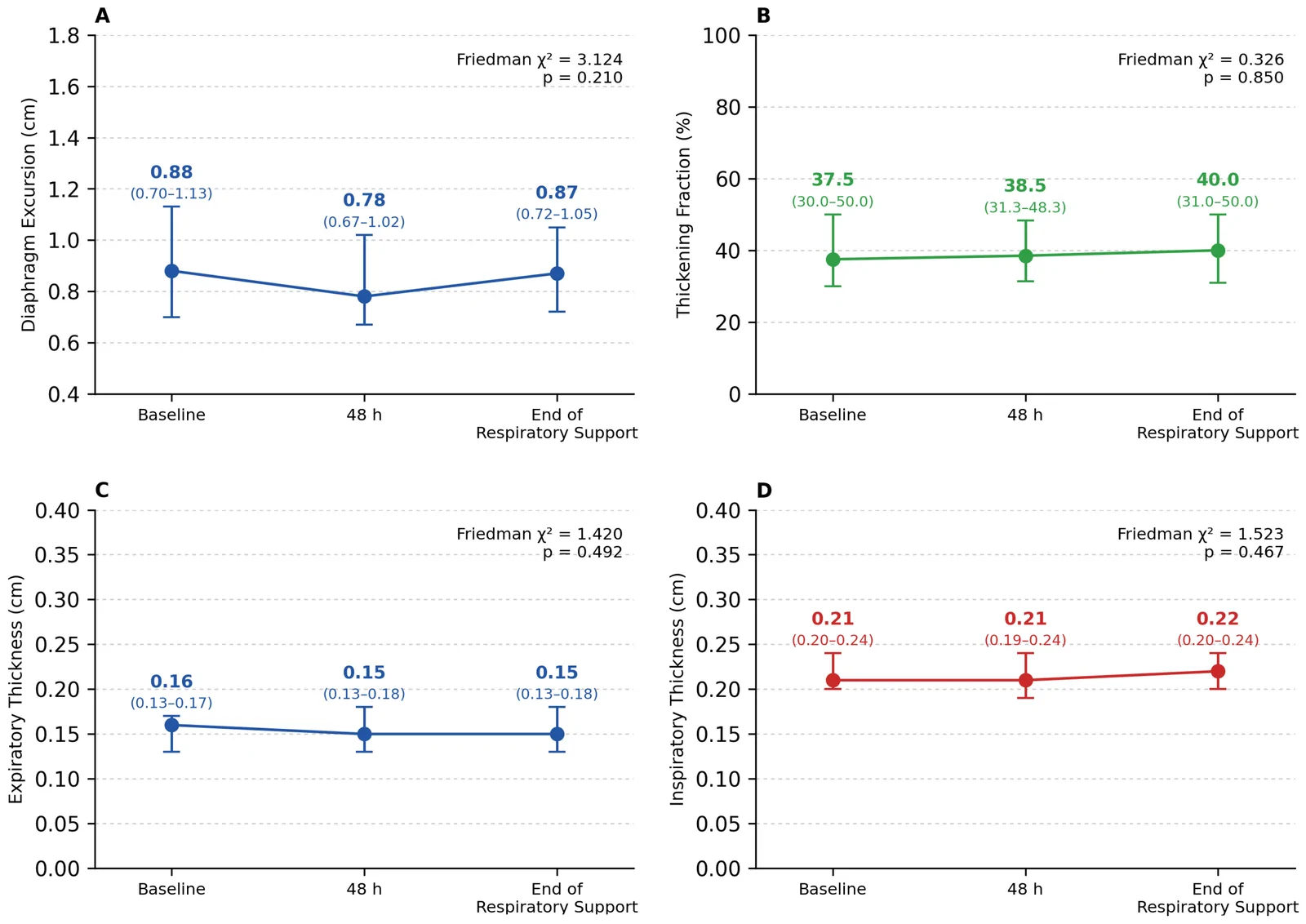
Serial changes in diaphragm ultrasound parameters during respiratory support. (**A**) Diaphragm excursion; (**B**) diaphragm thickening fraction; (**C**) end-expiratory diaphragm thickness; and (**D**) end-inspiratory diaphragm thickness at baseline, 48 h, and the end of respiratory support. Values are presented as medians with interquartile ranges. Longitudinal comparisons were performed using the Friedman test.

No statistically significant longitudinal changes in diaphragm ultrasound measurements were detected during respiratory support. Friedman analysis demonstrated no statistically significant longitudinal changes in DE (n = 100, χ^2^ = 3.124, *p* = 0.210), TFdi (n = 100, χ^2^ = 0.326, *p* = 0.850), DTee (n = 100, χ^2^ = 1.420, *p* = 0.492), or DTei (n = 100, χ^2^ = 1.523, *p* = 0.467) between baseline, 48 h, and the end of respiratory support (Figure 2).

Within the IMV subgroup, a sensitivity analysis was performed after excluding the five patients who underwent at least one ultrasound assessment during active neuromuscular blockade. No statistically significant longitudinal changes were detected in DE (n = 23, *p* = 0.316), TFdi (n = 23, *p* = 0.843), DTei (n = 23, *p* = 0.254), or DTee (n = 23, *p* = 0.397). These findings were consistent with the primary IMV subgroup analysis.

### 3.4. Relationship Between Diaphragm Ultrasound Changes and Respiratory Support Duration

No statistically significant relationships were observed between serial percentage changes in diaphragm ultrasound parameters and total respiratory support duration. Spearman correlation coefficients ranged from −0.006 to 0.089, and none reached statistical significance (Table 3).

Interobserver agreement for diaphragm ultrasound measurements was excellent, with intraclass correlation coefficients ranging from 0.89 to 0.96 (Supplementary Table S1).

Among invasively ventilated patients, ventilator settings at baseline and 48 h are summarized in Supplementary Table S2. Baseline DE was not significantly correlated with PEEP, PIP, mean airway pressure, tidal volume indexed to body weight, or FiO_2_ (all *p* > 0.05).

NIV failure occurred in 7 patients, and extubation failure occurred in 2 patients. No statistically significant differences in longitudinal diaphragm ultrasound changes were observed according to NIV failure (all *p* > 0.05). Because only two extubation failures occurred, comparisons according to extubation outcome were considered descriptive and were not interpreted as definitive.

## 4. Discussion

The present prospective study evaluated longitudinal changes in diaphragm ultrasound parameters in critically ill infants and children receiving different forms of respiratory support. No statistically significant longitudinal changes were detected in DE, DTee, DTei, or TFdi during short-term respiratory support. Furthermore, no statistically significant differences in baseline measurements or longitudinal percentage changes were detected according to the initial respiratory support modality. No statistically significant relationship was observed between serial diaphragm ultrasound changes and total respiratory support duration, while longitudinal changes did not differ significantly according to NIV failure; analyses according to extubation failure were limited by the low number of events. Together, these findings indicate that no clear longitudinal or between-group signal was identified in this cohort. However, because ventilatory assistance, spontaneous effort, sedation, and patient–ventilator interaction were not quantitatively standardized, the findings should not be interpreted as demonstrating physiological equivalence among respiratory support modalities.

The absence of significant longitudinal changes in diaphragm ultrasound parameters may reflect the complex interplay between respiratory muscle loading and unloading during respiratory support rather than the respiratory support modality itself. Experimental and clinical studies have shown that diaphragmatic injury may develop both when inspiratory effort is excessively suppressed, leading to disuse atrophy, and when respiratory muscle loading remains excessive despite ventilatory assistance, resulting in muscle fatigue and injury [6,7,17]. Accordingly, diaphragm-protective respiratory support aims to preserve an appropriate level of diaphragmatic activity rather than complete unloading or unrestricted spontaneous effort [6]. In the present cohort, no statistically significant longitudinal changes in DE, DTee, DTei, or TFdi were detected at the group level. Although the underlying physiological mechanisms cannot be determined from our data, the absence of a clear longitudinal signal may reflect multiple, simultaneously operating physiological and treatment-related factors. It should also be considered that the final ultrasound assessment was obtained immediately before discontinuation of respiratory support rather than at a fixed chronological interval. Consequently, the interval between baseline and the final assessment varied among patients, which may have influenced the interpretation of longitudinal changes.

Published pediatric reference data provide useful context for interpreting absolute diaphragm ultrasound measurements. In healthy infants and children, El-Halaby et al. reported that mean right diaphragmatic excursion increased with age, from approximately 0.64 cm in children aged 1 month–2 years to 1.31 cm in those aged 12–16 years [18]. In our cohort, children aged 1 month–2 years constituted 77% of the study population and had a mean baseline DE of 0.88 ± 0.28 cm, compared with 0.64 ± 0.21 cm in healthy children of the same age group [18]. Thus, DE was not uniformly low in the predominant younger subgroup of our cohort. This value was also broadly comparable with the mean DE of 10.39 ± 4 mm reported by Buonsenso et al. in infants with acute bronchiolitis [19], providing additional clinical context from a similarly young population with acute respiratory illness. Published pediatric reference values are also available for diaphragm thickness and thickening fraction; however, direct comparison across studies requires caution because the anatomical border definitions and measurement techniques used for diaphragm thickness are not standardized [18,20]. Because TFdi is derived from inspiratory and expiratory diaphragm thickness measurements, these methodological differences may also influence TFdi values.

However, findings across acute respiratory diseases are not uniform. Şık et al. reported that diaphragmatic excursion and thickness in children with pneumonia were lower than those reported in the youngest healthy reference group, which they interpreted as possible pneumonia-related diaphragmatic dysfunction. Nevertheless, within their pneumonia cohort, diaphragm excursion did not differ significantly across clinical severity groups, whereas thickening fraction decreased with increasing disease severity [21]. Taken together, these findings suggest that diaphragm excursion during acute respiratory illness does not follow a uniform pattern and may vary according to the underlying disease, age distribution, illness severity, and prevailing respiratory support conditions. Moreover, baseline measurements in our study were obtained within the first 6 h after initiation of respiratory support; therefore, early ventilatory assistance may have partially modified the observed excursion.

Unlike previous pediatric studies describing progressive diaphragm thinning during invasive mechanical ventilation [8,22,23], most children in our cohort had acute respiratory illnesses and relatively short periods of respiratory support. In addition, patients with chronic neuromuscular disorders, chronic ventilator dependence, and conditions expected to impair baseline diaphragm function were excluded. Only a small proportion of invasively ventilated patients received neuromuscular blockade, while spontaneous respiratory activity was preserved in patients receiving HFNC or NIV. These clinical characteristics may have limited prolonged diaphragmatic inactivity, which has been implicated in ventilator-induced diaphragm atrophy [16,22]. However, because respiratory effort, sedation depth, and patient–ventilator interaction were not quantitatively assessed, these mechanisms should be considered potential explanations rather than causal inferences.

Another important finding was that no statistically significant differences were detected in baseline diaphragm ultrasound measurements or longitudinal changes according to the initial respiratory support modality. Although HFNC, NIV, and IMV differ substantially in the degree of ventilatory assistance they provide, respiratory support modality alone may not accurately reflect the actual mechanical load imposed on the diaphragm. Accordingly, the absence of statistically significant between-group differences should not be interpreted as implying that HFNC, nasal NIV, mask NIV, and IMV exert equivalent physiological effects. Ventilator settings, spontaneous respiratory effort, patient–ventilator interaction, and the underlying respiratory disease may simultaneously influence diaphragmatic activity [6,17]. Therefore, the absence of statistically significant between-group differences in our cohort may reflect the combined influence of these factors rather than the respiratory support modality alone; however, this interpretation remains hypothetical because these determinants were not quantitatively standardized or measured. The predominantly negligible effect sizes indicate that no clear between-group signal was identified in this cohort; however, neither the nonsignificant comparisons nor the small effect sizes demonstrate physiological equivalence between respiratory support modalities.

No statistically significant relationship was detected between serial changes in diaphragm ultrasound parameters and total respiratory support duration or NIV failure. However, the extubation failure analysis was inconclusive because only two events occurred. Previous pediatric studies evaluating diaphragm ultrasonography have primarily examined measurements obtained immediately before extubation and have reported inconsistent findings regarding the predictive value of DE and TFdi for extubation success [9,10,11,12]. The recent systematic review by de Oliveira et al. likewise highlighted substantial heterogeneity among pediatric studies, including differences in patient populations, ultrasound protocols, and outcome definitions, limiting firm conclusions regarding the prognostic value of diaphragm ultrasonography [12]. However, the low number of failure events precludes firm conclusions regarding their prognostic utility.

The present findings have several potential clinical implications. First, they provide prospective longitudinal data describing diaphragm ultrasound behavior across the full spectrum of respiratory support modalities used in PICUs, including HFNC, NIV, and IMV, within a standardized assessment protocol. Second, no statistically significant short-term longitudinal changes were detected in this cohort; however, the study was not designed to establish the expected magnitude of change in all critically ill children. Finally, because no statistically significant relationships with respiratory support duration or NIV failure were detected and the number of failure events was limited, diaphragm ultrasound measurements should be interpreted as one component of the overall clinical assessment rather than as an isolated prognostic marker. Whether serial diaphragm ultrasonography may provide greater clinical value in selected high-risk populations or during prolonged respiratory support warrants further investigation.

Several limitations should be acknowledged. First, this was a single-center study including a heterogeneous population of critically ill children with different underlying causes of acute respiratory failure, which may limit the generalizability of the findings. The study population was predominantly composed of infants and young children, and bronchiolitis or pneumonia accounted for 89% of admission diagnoses. Therefore, the present findings primarily reflect young children with acute respiratory infections rather than the full spectrum of critically ill pediatric patients. In addition, exclusion of patients in whom serial ultrasound assessments could not be completed may have introduced selection bias, because these patients may have differed from those included in the complete-case analyses in terms of illness severity, clinical instability, or respiratory support duration. Second, although pediatric longitudinal studies of diaphragm ultrasonography were available, they did not provide directly comparable effect-size estimates across HFNC, NIV, and IMV; therefore, a formal a priori sample-size calculation could not be reliably performed. Although all consecutive eligible patients were prospectively enrolled during the predefined study period, the study may have been underpowered to detect small between-group differences or modest relationships with clinical outcomes. Accordingly, the absence of statistically significant findings should not be interpreted as evidence of equivalence between respiratory support modalities. Third, the respiratory support groups were not randomized, and the initial support modality was selected according to clinical need. Consequently, unmeasured confounding by indication may have influenced between-group comparisons. Fourth, the numbers of NIV failures and extubation failures were low, limiting the statistical power of these exploratory analyses. Fifth, respiratory effort, sedation depth, patient–ventilator interaction, and the level of ventilatory assistance were not quantitatively assessed and therefore could not be incorporated into the interpretation of diaphragm ultrasound findings. Although all serial examinations were performed by the same experienced operator to reduce interoperator variability, this approach may have introduced operator-dependent measurement bias and may limit the generalizability of the findings. Nevertheless, interobserver reliability was excellent in the subset of patients independently assessed by a second blinded observer. Finally, only the right hemidiaphragm was evaluated, consistent with current ultrasound recommendations favoring the right side because of its superior acoustic window; therefore, bilateral diaphragm function was not assessed. In addition, serial ultrasound measurements were confined to the period of respiratory support, and no follow-up assessments were performed after PICU discharge.

## 5. Conclusions

No statistically significant longitudinal changes in diaphragm ultrasound parameters were detected during short-term respiratory support, and no statistically significant differences were identified among the initial respiratory support groups. These findings should not be interpreted as demonstrating physiological equivalence between respiratory support modalities. Serial diaphragm ultrasonography should be interpreted within the broader clinical context rather than as an isolated prognostic tool. Further multicenter studies with larger and more diverse pediatric populations are needed to clarify the clinical utility and prognostic value of serial diaphragm ultrasonography during respiratory support.

## Figures and Tables

**Table 1:** Baseline characteristics of the study population.

Characteristic	N = 100
Age, months, median (IQR)	4 (2–24)
Male sex, n (%)	66 (66.0)
Weight, kg, median (IQR)	7 (5–13)
Primary diagnosis at PICU admission, n (%)	
Bronchiolitis/pneumonia	89 (89.0)
Neurological diseases	5 (5.0)
Trauma	4 (4.0)
Cardiac diseases	2 (2.0)
Presence of comorbidity, n (%)	6 (6.0)
Type of respiratory support, n (%) *	
HFNC	31 (31.0)
Nasal NIV	17 (17.0)
Mask NIV	24 (24.0)
IMV	28 (28.0)
Neuromuscular blockade during IMV, n (%) †	5 (17.8)

* Initial respiratory support at PICU admission; † Percentage calculated among patients receiving invasive mechanical ventilation (5/28); IQR, interquartile range; PICU, pediatric intensive care unit; HFNC, high-flow nasal cannula; NIV, noninvasive ventilation; IMV, invasive mechanical ventilation.

**Table 2:** Comparison of baseline and longitudinal diaphragm ultrasound parameters according to the initial respiratory support modality.

Variable	HFNC	Nasal NIV	Mask NIV	IMV	*p*
Baseline					
DE (cm)	0.83 (0.70–1.00)	0.78 (0.58–1.13)	0.90 (0.73–1.18)	0.90 (0.64–1.18)	0.594
TFdi (%)	33.3 (25.0–50.0)	33.0 (29.5–41.6)	37.9 (31.6–44.8)	40.5 (30.9–53.7)	0.410
DTee (cm)	0.16 (0.13–0.18)	0.16 (0.14–0.23)	0.16 (0.13–0.17)	0.15 (0.13–0.17)	0.442
DTei (cm)	0.21 (0.18–0.24)	0.24 (0.18–0.29)	0.21 (0.18–0.24)	0.21 (0.19–0.24)	0.529
Early change (Baseline–48 h)					
%ΔDE	0 (−12.9–1.2)	0 (−31.6–22.5)	−1.0 (−15.2–3.3)	0 (−3.4–6.1)	0.606
%ΔTFdi	7.5 (−18.4–38.7)	17.0 (−13.3–49.9)	0 (−20.0–24.9)	−3.3 (−29.3–24.2)	0.274
%ΔDTee	5.3 (−7.8–15.3)	−3.1 (−27.0–6.2)	−0.62 (−11.7–8.3)	0 (−9.7–7.0)	0.416
%ΔDTei	0 (−4.9–20.2)	−3.1 (−20.8–8.8)	0 (−13.0–5.8)	−4.4 (−15.4–5.4)	0.175
Change from baseline to end of respiratory support					
%ΔDE	0 (−10.8–10.5)	13.3 (−25.9–40.4)	0 (−13.7–9.9)	6.0 (−4.2–24.2)	0.321
%ΔTFdi	0 (−12.5–61.5)	13.3 (−23.2–56.2)	4.0 (−11.0–44.8)	0.7 (−28.5–37.1)	0.685
%ΔDTee	0 (−10.0–11.1)	−8.3 (−20.9–0)	0 (−12.3–8.0)	−2.2 (−13.8–15.3)	0.540
%ΔDTei	0 (−9.0–16.6)	0 (−15.6–5.3)	0 (−9.5–11.7)	−4.7 (−12.5–13.4)	0.452

Values are presented as median (IQR). Between-group comparisons were performed using the Kruskal–Wallis test; Percentage change was calculated as ((follow-up − baseline)/baseline) × 100. Early change was calculated using the 48-h measurement, whereas end-of-respiratory-support change was calculated using the final diaphragm ultrasound measurement obtained immediately before discontinuation of respiratory support; DE, diaphragm excursion; TFdi, diaphragm thickening fraction; DTee, end-expiratory diaphragm thickness; DTei, end-inspiratory diaphragm thickness; HFNC, high-flow nasal cannula; NIV, noninvasive ventilation; IMV, invasive mechanical ventilation; IQR, interquartile range.

**Table 3:** Exploratory relationships between serial diaphragm ultrasound changes and respiratory support duration.

Ultrasound Parameter	Spearman’s ρ	*p* Value
Early %ΔDE	0.053	0.639
Early %ΔTFdi	−0.006	0.955
Early %ΔDTee	0.054	0.624
Overall %ΔDE	0.089	0.377
Overall %ΔTFdi	0.018	0.862
Overall %ΔDTee	0.089	0.376

Data were analyzed using Spearman’s rank correlation coefficient. No statistically significant relationships were observed between serial diaphragm ultrasound changes and total respiratory support duration.

## Data Availability

The data supporting the findings of this study are available from the corresponding author upon reasonable request. The data are not publicly available because they contain information that could compromise participant privacy.

## Supplementary Materials

The following supporting information can be downloaded at https://www.mdpi.com/article/10.3390/children13081104/s1, Table S1: Interobserver reliability of diaphragm ultrasound measurements; Table S2: Invasive mechanical ventilation settings at baseline and the 48-h diaphragm ultrasound assessment; Table S3: Effect sizes for comparisons of diaphragm ultrasound parameters according to the initial respiratory support modality.
