# Supplementary material for: Longitudinal Diaphragm Ultrasound Assessment Across Respiratory Support Modalities in Critically Ill Infants and Children: A Prospective Cohort Study
Source: Children (Basel). 2026 Aug 18;13(8):1104. doi: 10.3390/children13081104 (PMC13511676; doi:10.3390/children13081104)
Supplement: Supplementary file 1 [file children-13-01104-s001.zip › children-4474638-supplementary.pdf]

Supplementary Table S1. Interobserver reliability of diaphragm ultrasound measurements.

**Ultrasound parameter**	**ICC (95% CI)**
Diaphragm excursion (DE)	0.96 (0.84–0.99)
End-expiratory diaphragm thickness (DTee)	0.94 (0.79–0.99)
End-inspiratory diaphragm thickness (DTei)	0.92 (0.74–0.98)
Diaphragm thickening fraction (TFdi)	0.89 (0.65–0.97)

Supplementary Table S2. Invasive mechanical ventilation settings at baseline and the 48-h diaphragm ultrasound assessment.

Setting	Baseline	48 h
PIP, cmH_2O	25 (20-30)	27 (24-31)
PEEP, cmH_2O	7 (6-8)	8 (7-8)
MAP, cmH_2O	14.5 (11.75-16.5)	15 (14-16)
Tidal volume, mL/kg	9.7 (7.3-10.4)	10.4 (8.3-14.1)
FiO_2, %	60 (50-60)	50 (40-60)

Values are presented as median (interquartile range).

PIP, peak inspiratory pressure; PEEP, positive end-expiratory pressure; MAP, mean airway pressure; FiO_2, fraction of inspired oxygen.

Supplementary Table S3. Effect sizes (epsilon squared) for comparisons of diaphragm ultrasound parameters according to the initial respiratory support modality.

**Variable**	**Kruskal–Wallis H**	**ε^2**	**Interpretation***
**Baseline**			
DE	1.898	0.000	Negligible
TFdi	2.884	0.000	Negligible
DTee	2.692	0.000	Negligible
DTei	2.214	0.000	Negligible
**Early change (Baseline–48 h)**			
%ΔDE	1.841	0.000	Negligible
%ΔTFdi	3.888	0.011	Small
%ΔDTee	2.844	0.000	Negligible
%ΔDTei	4.956	0.024	Small
**Change from baseline to end of respiratory support**			
%ΔDE	3.498	0.005	Negligible
%ΔTFdi	1.488	0.000	Negligible
%ΔDTee	2.158	0.000	Negligible
%ΔDTei	2.629	0.000	Negligible

*Interpretation based on conventional epsilon squared thresholds:

- <0.01 = negligible
- 0.01–0.06 = small
- 0.06–0.14 = moderate
- ≥0.14 = large

Intraclass correlation coefficients were calculated using a two-way random-effects, absolute-agreement, single-measure model.
